# The phylogeny of brown lacewings (Neuroptera: Hemerobiidae) reveals multiple reductions in wing venation

**DOI:** 10.1186/s12862-016-0746-5

**Published:** 2016-09-20

**Authors:** Ivonne J. Garzón-Orduña, Imelda Menchaca-Armenta, Atilano Contreras-Ramos, Xingyue Liu, Shaun L. Winterton

**Affiliations:** 1 California Department of Food & Agriculture, California State Collection of Arthropods, 3294 Meadowview Rd, Sacramento, CA USA; 2 Instituto de Ciencias Básicas e Ingeniería, Universidad Autónoma del Estado de Hidalgo, Pachuca, Hidalgo Mexico; 3 Departamento de Zoología, Instituto de Biología, UNAM, Ciudad Universitaria, México, DF Mexico; 4 Department of Entomology, China Agricultural University, Beijing, 100193 China

## Abstract

**Background:**

The last time the phylogenetic relationships among members of the family Hemerobiidae were studied quantitatively was over 12 years ago and based exclusively on morphology. Our study builds upon this morphological evidence by adding sequence data from three gene loci to provide a total evidence phylogeny of brown lacewings (Neuroptera: Hemerobiidae). Thirty-seven species representing nineteen Hemerobiidae genera were compared with outgroups from the families Ithonidae, Psychopsidae and Chrysopidae in Bayesian and parsimony analyses using a single nuclear gene (CAD) and two mitochondrial (16S rDNA and Cytochrome Oxidase I) genes. We compare divergence time estimates of Hemerobiidae cladogenesis under the two most commonly used relaxed clock models and discuss the evolution of wing venation in the family.

**Results:**

We recovered a phylogeny largely incongruent with previously published morphological studies, although all but two subfamilies (i.e., Notiobiellinae and Drepanacrinae) were recovered as monophyletic. We found the subfamily Drepanacrinae paraphyletic with respect to Psychobiellinae, and Notiobiellinae to be polyphyletic. We thus offer a revised concept of Notiobiellinae, comprising only *Notiobiella* Banks, and erect a new subfamily Zachobiellinae including the remaining genera previously placed in Notiobiellinae. Psychobiellinae is synonymized with Drepanacrinae. Unlike the previous hypothesis that proposed a remarkably laddered topology, our tree suggests that hemerobiids diverged as three main clades. Moreover, in contrast to the vein proliferation hypothesis, we found that hemerobiids have instead undergone multiple reductions in the number of radial veins, this scenario questions the relevance of this character as diagnostic of various subfamilies

**Conclusions:**

Our phylogenetic hypothesis and divergence times analysis suggest that extant hemerobiids originated around the end of the Triassic and evolved as three distinct clades that diverged from one another during the Late Jurassic to Early Cretaceous. Contrary to earlier phylogenetic hypotheses, *Carobius* Banks (Carobiinae) is sister to the previously unplaced genus *Notherobius* New in a clade more closely related to Sympherobiinae, Megalominae and Zachobiellinae subfam. nov. The addition of taxa which are not available for DNA sequencing should be the focus of future studies, especially *Adelphohemerobius* Oswald, which is particularly important to test our inferences regarding the evolution of wing venation in Hemerobiidae.

**Electronic supplementary material:**

The online version of this article (doi:10.1186/s12862-016-0746-5) contains supplementary material, which is available to authorized users.

## Background

**Fig. 1:**
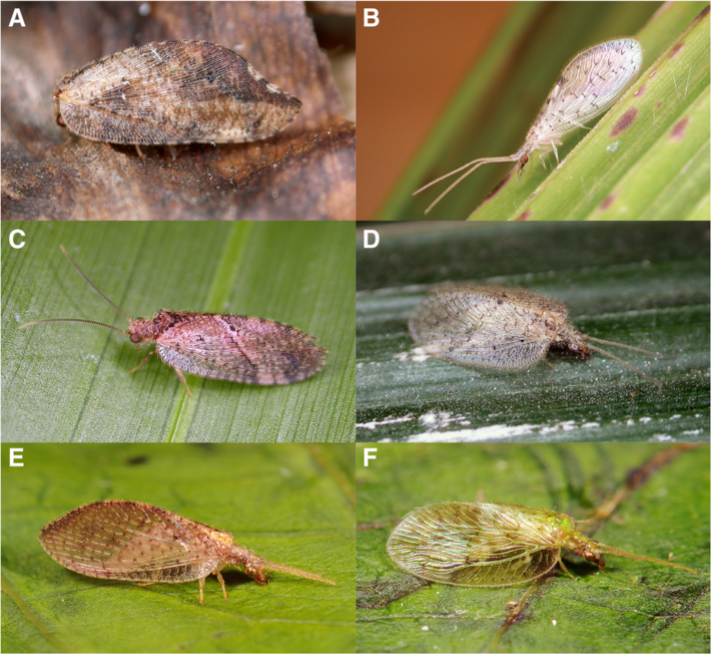
Representatives of adult brown lacewings (Hemerobiidae). **a** *Drepanacra binocula* (Newman), Australia; **b** *Zachobiella pallida* Banks, Australia; **c** *Megalomus pictus* Hagen, Costa Rica; **d** *Psectra nakaharai* New, Australia; **e** *Hemerobius incursus* Banks, Malaysia; **f** *Notiobiella nguyeni* Makarkin, Malaysia. (Photographs A–D copyright Shaun L. Winterton, E–F copyright Stephen D. Gaimari)

Hemerobiidae (brown lacewings), as their common name suggests, are relatively small lacewings with brown wings and body (Fig. 1). Most species are rather cryptic (e.g., Fig. 1a), nocturnally active [1] and often feign death when disturbed [2]. Not all hemerobiids are brown though, as some species in the genus *Notiobiella* Banks are green (Fig. 1f). Representatives of the family are found on all continents except for Antarctica [3]. Genera such as *Hemerobius* Linnaeus and *Micromus* Rambur are almost cosmopolitan, while other genera are geographically restricted to particular continents, such as *Carobius* Banks*, Notherobius* New and *Psychobiella* Banks in Australia, and *Conchopterella* Handschin and *Nomerobius* Navás in South America*.* Tauber et al. [4] offer a detailed summary of the natural history of members of this family, which is predominantly as arboreal predators in both adult and larval stages.

Recent quantitative phylogenetic analyses of family level relationships among lacewings have confirmed that Hemerobiidae and Chrysopidae (green lacewings) are sister groups (e.g., [5], [6]). Other features aside from body size and color differentiate hemerobiids from chrysopids. Hemerobiids most notably have wings with multiple radial vein sectors and trichosors present (i.e., small thickenings of the wing margin between the tips of veins and veinlets) [7]. Brown and green lacewing larvae are both arboreal predators of phytophagous pests (e.g., aphids, mites, mealy bugs) and therefore are considered important as biological control organisms [8]. Hemerobiid larvae can be readily differentiated from chrysopid larvae as they lack a trumpet-shaped empodium typical of second and third instars of Chrysopidae; they also do not have setiferous tubercles on the thorax and abdomen and thus do not carry a debris-packet [9]. Chrysopid eggs are laid on silken stalks, whereas hemerobiid eggs are laid singly (or in small groups) on the substrate [1].

Oswald [3] presented the first comprehensive taxonomic revision of the family with a detailed phylogenetic analysis of genus-level relationships to erect the present subfamilial classification of Hemerobiidae. This analysis included 24 of the 25 recognized genera of extant Hemerobiidae at the time, which were scored for 107 morphological characters. Subsequent papers by Oswald [10, 11] added two new genera, one of which was placed in its own subfamily. In Oswald’s hypothesis, the phylogenetic relationships among subfamilies are completely imbalanced (i.e., laddered relationship), with the monotypic subfamily Adelphohemerobiinae as the sister lineage to the rest of Hemerobiidae, Carobiinae is sister to the remaining subfamilies excluding Adelphohemerobiinae, Hemerobiinae is sister to the rest excluding Carobiinae and Adelphohemerobiinae, etc. (Inset in Fig. 3) [3, 10, 11].

**Fig. 2:**
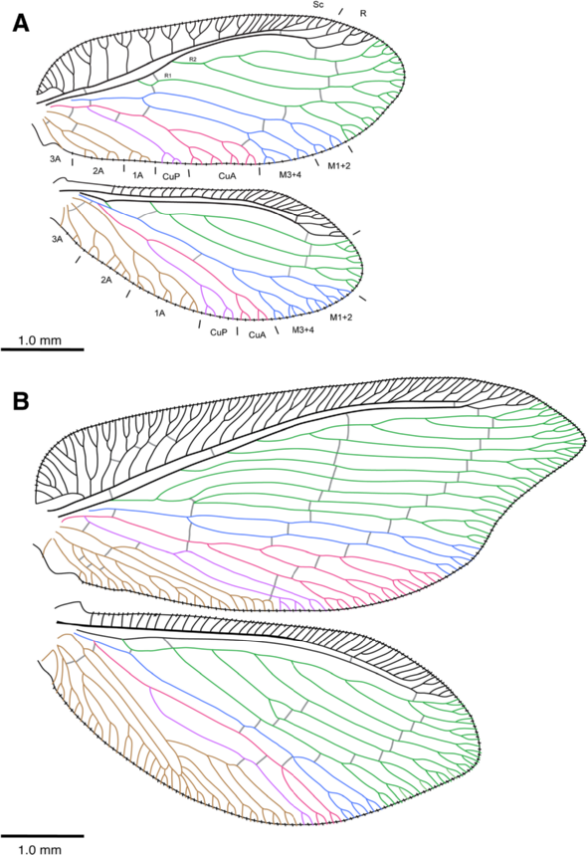
Hemerobiidae wing venation: **a** *Carobius pulchellus* Banks; **b** *Drepanacra binocula* (Newman) (after Oswald [3])*.* Wing venation colors correspond to different wing veins (Green: radial, blue: medial, pink: anterior cubitus, purple: posterior cubitus, brown: anal veins). Vestiture omitted

Currently, Hemerobiidae includes approximately 560 species [12, 13) divided among 10 subfamilies. The subfamilies are diagnosed mainly by features of the wing venation and genitalic morphology [3, 10, 11]. The multiple radial veins (Rs) originating on R1 (=oblique radial branches (ORB’s) sensu Oswald [3]) in the forewing (Fig. 2) is considered synapomorphic for the family and deviates from the condition found in all other extant Neuroptera, whose forewings have only one radial sector; multiple radial sectors are found also in some extinct lacewings, e.g., some Kalligrammatidae (Mesozoic). The number of radial veins in Hemerobiidae forewings varies considerably, ranging in some genera with only two (e.g., *Carobius* Banks) to 13 veins in the genus *Drepanepteryx* Leach [3]. However, the most common condition is the presence of two to three radial veins (e.g., Fig. 2a). Other synapomorphies for Hemerobiidae include galea bearing penicilliform sensilla, clypeus bearing several pairs of primary setae, and female insemination-fertilization canal opening of pore-entry type (sensu Oswald [3]).

**Fig. 3:**
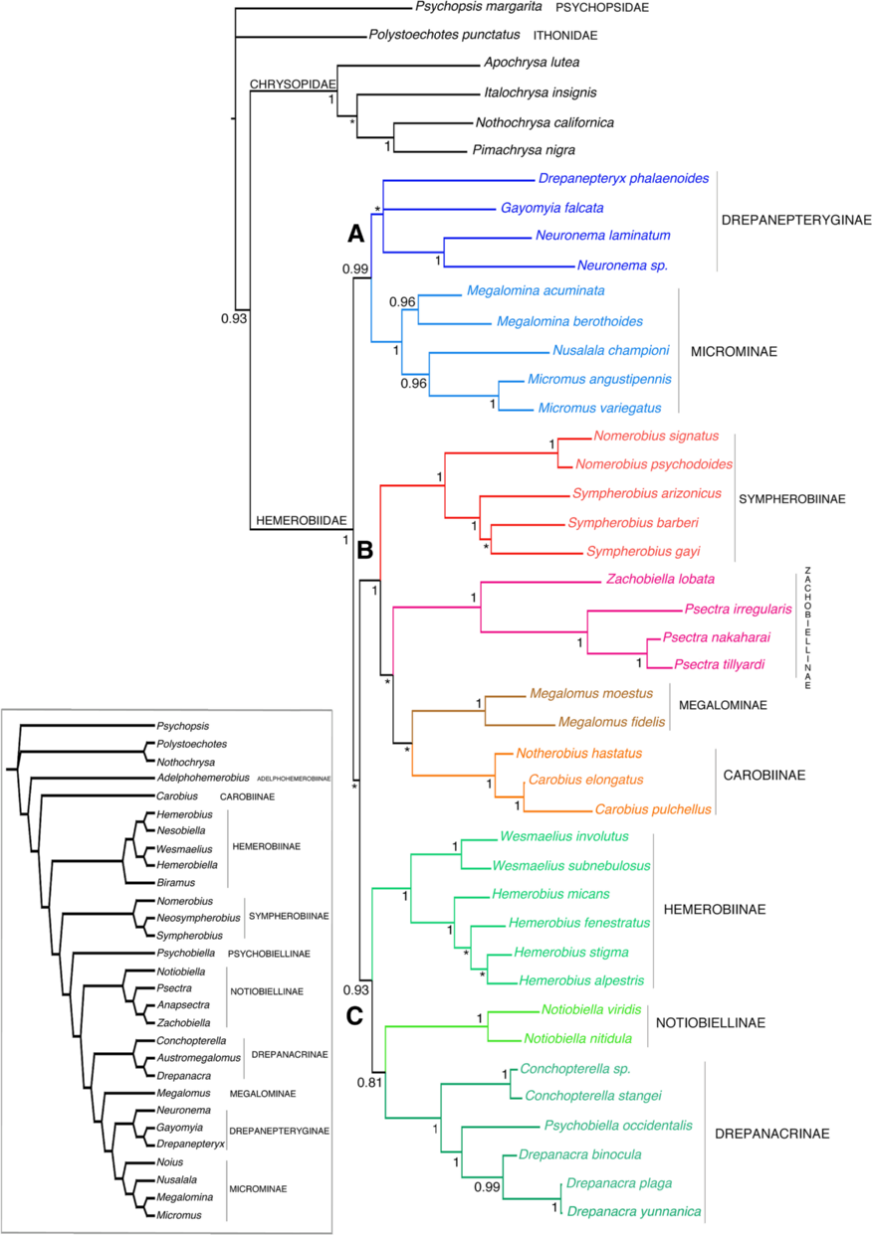
Phylogeny of Hemerobiidae based on Bayesian analysis of the combined data of 101 morphological characters and DNA from three molecular fragments (2760 bp). Posterior probability (PP) values are reported in front of each corresponding node; an asterisk denotes a node with PP support less than 0.81. To ease comparison with our topology, we provide in the inset the phylogenetic relationships in Hemerobiidae after Oswald [3, 10, 11] based on morphological characters

The importance of Oswald’s [3, 10, 11] phylogenetic studies cannot be understated; these works not only provided the diagnostic characters used today to identify most of the genera, they also are the first quantitative analyses and, to date, the only substantive hypotheses regarding hemerobiid intergeneric relationships (Inset in Fig. 3). Our study builds upon Oswald’s phylogenetic and taxonomic studies by adding mitochondrial and nuclear molecular data to his morphological matrix. Herein, we present a phylogeny of Hemerobiidae based on the combination of DNA sequence data for multiple loci with morphological scoring. We also present an estimate of divergence times on a geological time scale.

## Methods

### Exemplar selection

Our study includes 37 species that represent 19 of the 30 described genera of extant Hemerobiidae. Although limited in the number of species, our efforts were directed to cover the biological diversity and taxonomic breath of a family of organisms that are uncommonly collected. Notwithstanding, most genera (13) sampled here included more than one species. We used as out-groups species in the closely related families Ithonidae (*Polystoechotes* Burmeister), Psychopsidae (*Psychopsis* Newman) and Chrysopidae (*Nothochrysa* McLachlan, *Apochrysa* Schneider, *Italochrysa* (Walker) and *Pimachrysa* Adams), the latter being the sister family of Hemerobiidae [6]. Except for the additional chrysopids, these outgroups correspond to the genera (and in most cases even the species) used in the previous published morphological phylogenetic analysis of the family by Oswald [3]. The genus *Berothimerobius* Monserrat & Deretsky was not included in the analysis as this genus, although originally described in Hemerobiidae, was subsequently synonymized with *Ormiscocerus* Blanchard in Gay and placed in Berothidae [14]. Vouchers of all specimens sequenced are deposited in the California State Collection of Arthropods in Sacramento, USA (Table 1). Genera included in the analysis include *Carobius*, *Conchopterella*, *Drepanacra* Tillyard, *Drepanepteryx*, *Gayomyia* Banks, *Hemerobius*, *Megalomus* Rambur, *Micromus*, *Megalomina* Banks, *Neuronema* McLachlan, *Nomerobius*, *Notherobius*, *Notiobiella*, *Nusalala* Navás, *Psectra* Hagen, *Psychobiella*, *Sympherobius* Banks, *Wesmaelius* Krüger and *Zachobiella* Banks. Some genera were not available for DNA sequencing; genera absent from this analysis are *Adelphohemerobius* Oswald, *Anapsectra* Tjeder, *Austromegalomus* Esben-Petersen, *Biramus* Oswald, *Hemerobiella* Kimmins, *Nesobiella* Kimmins, *Neosympherobius* Kimmins, and *Noius* Navás. In many cases, a closely related sister genus was available as a surrogate for phylogenetic placement, for example, *Hemerobius* for *Hemerobiella*, *Sympherobius* for *Neosympherobius*, and *Micromus* for *Noius*. Unfortunately, important putative adelphotaxa either for the family (i.e., *Adelphohemerobius*; sensu Oswald [11]) or particular subfamilies (i.e., *Biramus*, sensu Oswald [10]) were missing from the analyses without suitable sister group analogs.

**Table 1:** List of specimens included in this study

Taxon	Genbank accession numbers			Voucher code	Voucher collection data/source
	16S	COI	CAD		
PSYCHOPSIDAE: *Psychopsis margarita* Tillyard	EU734897	EU839764*	EU860149	PSYP CASENT8092209 in Winterton et al. [6]	AUSTRALIA: Queensland: Brigalow Res. Stn., 27–28.×.2000, Queensland Museum party [9804] 24°48′S, 149°45′E
POLYSTOECHOTIDAE: *Polystoechotes punctatus* (Fabricius)	EU734893	EU839760	EU860146	POLY CASENT8092171 in Winterton et al. [6]	USA: Idaho: Latah Co., Moscow, 19.viii.2001, J.B. Johnson
CHRYSOPIDAE: *Nothochrysa californica* Banks	DQ399283	DQ414505*	EU860135	NOTH CASC205 in Winterton et al. [6]	USA: California: Monterey Co., Pfeiffer Big Sur, 2.iii.2003, J. & A. Skevington [36° 14.939′N, 121° 46.466′W]
*Italochrysa insignis* (Walker)	DQ399283	DQ414485	EU860117	ITAL CASC210 in Winterton et al. [6]	AUSTRALIA: Queensland: Brisbane, Mt. Coot-tha, 14.i.2000, S.L. Winterton [27° 28.574′S, 152° 57.817′E]
*Pimachrysa nigra* Adams	EU734889	EU839756	EU860142	PIMA CASENT8092214 in Winterton et al. [6]	USA: California: Sacramento Co., Pine Hill, 24.iii.2003, J. Skevington, 38° 43′N, 120° 59′W.
*Apochrysa lutea* (Walker)	DQ399285	EU839753	EU860139	APO CASC203 in Winterton et al. [6]	AUSTRALIA: Queensland: Brisbane, 13.xii.1998, S.L. Winterton [27° 28.574′S, 152° 57.817′E]
HEMEROBIIDAE:					
*Carobius elongatus* New	KX223365	–	KX247653	CAREL	AUSTRALIA: New South Wales: Girralang Nature Reserve, 20.6 km NE Orange, Malaise, 15–18iii.2002, C.L. Lambkin, N. Starick, 33°09′21″S, 149°15′11″E
*Carobius pulchellus* Banks	KX223366	KX060787*	KX247654	CAPUL	AUSTRALIA: Queensland: Brisbane, 12.xii.1999, S.L. Winterton
*Conchopterella* sp*.*	KX223367	KX085005*	KX247655	CONSP	CHILE: Region IV, Limari Province: Fray Jorge National Park, Quebrada Honda I, malaise trap in wash, 1–7.x.2003, M.E. Irwin, F.D. Parker, 122 m, -30° 41.4′, 71° 37.8′
*Conchopterella stangei* (Gonzalez-Olazo)	EU734855	DQ414494	DQ414474	CONC CASC202 in Winterton et al. [6]	CHILE: Osorna Prov.: Agua Calientes, Puyehue N.P., 1–5.xii.2003, M.E. Irwin, 40°43.94′S, 72°18.83′W
*Drepanacra binocula* (Newman)	KX223368	KX085006*	KX247656	DREPAC	AUSTRALIA: New South Wales: Pilliga Scrub, -31.821, 149.473, 28.x.2008, S.L. Winterton, dry creek bed
*Drepanacra yunnanica* Yang	KX223369	KX085007	–	DRETH	THAILAND: Chiang Mai, Doi Phahompok NP. Kiewlom1: Montane Forest. 20° 03.455′N 99°08.551 E. 2174 m. Malaise trap, 7–14.ix.2007. Komwuan Srisom & Prasit Wongchai. T2810.
*Drepanacra plaga* Banks	KX223370	KX085008*	–	DREPATH	THAILAND: Chiang Mai, Doi Inthanon NP, checkpoint 2. 18°31.554′ N. 98°29.940′E 1700 m. Malaise trap 1–8.xii.2006. T1876.
*Drepanepteryx phalaenoides* (Linnaeus)	EU734861	EU839731*	EU860113	DREPH CASENT8092207 in Winterton et al. [6]	GREECE: Peloponnisos Messinia, Kardamili, 31.v.2000, K.C. Holston, 26°54′N, 22°14′E
*Gayomyia falcata* (Blanchard)	KX223371	KX085009	–	GAYOM	CHILE: Region X, Osorno Province: Aguas Calientes, Puyehue National Park, Malaise trap in *Nothofagus* forest, 1–5.xi.2003, M.E. Irwin, 1253 m, -40° 43.94′, 72° 18.83′
*Hemerobius alpestris* Banks	KX223372	KX085010 *	KX247657	HEMAL	USA: New Mexico: Cloudcroft, August 2001, S.L. Winterton & J.D. Oswald
*Hemerobius fenestratus* Tjeder	AY620147	–	–	HEMERFEN HEMFEN1 in Haring and Aspöck [5]	AUSTRIA: Dürnstein
*Hemerobius micans* olivier	KX223373	KX085011 *	KX247658	HMICAN	ITALY: Abruzzo (AO) National Park, Val Fondillo, 10.vii.1999, A. Letardi
*Hemerobius stigma* Stephens	KX223374	KX085012 *	KX247659	HEMST	USA: New Mexico: Cloudcroft, August 2001, S.L. Winterton & J.D. Oswald
*Megalomina acuminata* Banks	KX223375	KX085013 *	–	MEGAC	AUSTRALIA: Queensland: Brisbane Forest Park, Scrub Road, -27.427, 152.841, 13.xii.2007, Malaise in rainforest, S.L. Winterton, J.S. Bartlett
*Megalomina berothoides* (McLachlan)	KX223376	KX085014 *	–	MEGBE	AUSTRALIA: Western Australia: Cliff Head, 20.ix–9.xi.2003, C.L. Lambkin, N. Starick, J. Recsei, Malaise in Mallee, 29°31′33″S, 114°59′44″E
*Megalomus moestus* Banks	KX223377	KX085015	–	MEGLM	USA: Utah: Provo, T. Waite, 8.viii.2000
*Megalomus fidelis* (Banks)	KX223378	–	KX247660	MEGSP	USA: North Carolina, 2001. Det. B. Wiegmann.
*Micromus variegatus* (Fabricius)	KX223379	–	–	MICKO	SOUTH KOREA: Jirisan: Hamyang-gum, Macheon-myon Samjeong-li, 8.v–5.vi.2004, 35° 20.930, 127° 38.503, Tripotin coll. Malaise
*Micromus angustipennis* (Perkins)	KX223380	KX085016 *	–	MICHW	USA: Hawaii: Alaki Swamp, NaPaii Kona Forest Res., 18.viii.2006, D. Rubinoff, G. Eiben, UV light
*Neuronema laminatum* Tjeder	KX223381	KX085017	–	NENEM	CHINA: HeShangPu forest, Ningxia province, 2130 m. Yang Zhao, 2012-viii-12. Cau Num3.
*Neuronema sp.*	KX223382	KX085018	–	NEUNM	THAILAND: Chiang Mai, Doi Phahompok NP. Kiewlom1: Montane Forest. 20° 03.455′N 99°08.551 E. 2174 m. Malaise trap, 7–14.ix.2007. Komwuan Srisom & Prasit Wongchai. T2815.
*Nomerobius signatus* (Hagen)	KX223383	–	KX247661	NOMER	CHILE: Quillota Province: Las Palmas de Ocoa, malaise in hillside draw, 215 m, 2–10.i.2000, M.E. Irwin, E.I. Schlinger, -32.9324° 71.6781°
*Nomerobius psychodoides* (Blanchard)	KX223384	–	–	NOMSP	CHILE:Quillota Prov. Las palmas de Ocoa. Irwin & Schlinger, 2–10, i. 2000
*Notherobius hastatus* New	KX223385	KX085019 *	KX247662	NOTHA	AUSTRALIA: New South Wales: Kosciuszko National Park, 1.7 km ENE Thredbo, 6–15.iii.2003, 1380 m C. Lambkin, N. Starrick, J. Recsei, Malaise over narrow creek, 36°30′07″S, 148°19′02″E
*Notiobiella viridis* Tillyard	EU734883	EU839750	EU860136	NOTICASENT8092205 in Winterton et al. [6]	AUSTRALIA: Queensland: Rockhampton, 29.i.2000, S.L.Winterton [23°18.754′S, 150° 30.966′E]
*Notiobiella nitidula* Navás	KX223386	KX085020	KX247663	NOTIMG CASENT3006314	MADAGASCAR: Mahajanga Province: Namoroka National Park, 17.8 km WNW Vilanandro, 100 m, 8–12.xi.2002, 16° 22′36″S, 45° 19′36″ Fisher, Griswald, et al., at light
*Nusalala championi* Kimmins	KX223387	KX085021 *	–	NUSAC	PERU: Pasco, Yanachaga-Chemillen N.P., San Alberto Valley, 2,300 m, 10–13.x.2002, 10°32′39.7″S, 75°22′00.1″W, Malaise across stream, D. Takiya, C. Pena, R. Rakitov
*Psectra nakaharai* New	KX223388	KX085022	KX247664	PSECN	AUSTRALIA: Victoria: Bendoc-Bonag State Forest, 61 km NNE Orbost, Malaise, 11.i–12.ii.2005, C.Lambkin, N. Starick, 37°12′31″S, 148°44′01″E
*Psectra tillyardi* (Kimmins)	KX223389*	–	KX247665	PSETI	AUSTRALIA: New South Wales: Warrumbungle National Park, Buckley’s Creek, -31°16.083, 149°00.344, 398 m, 13.iii.2008, S.L Winterton, J.S. Bartlett, D.J. Tree, Malaise across creek bed
*Psectra* na *irregularis* (Carpenter)	KX223390	–	–	PSENC	NEW CALEDONIA: Sud Province: 9.3 km NW Sarramea, [-21.581, 165.787], 497 m, 17–24.xi.1998, Malaise trap, M.E. Irwin, E.I. Schlinger,
*Psychobiella occidentalis* New	KX223391	KX085023 *	KX247666	PSYOCC	AUSTRALIA: Western Australia: Rocky Gully, -34.509, 117.113, 19.xi.2008, roadside vegetation, S.L. Winterton & S.D. Gaimari
*Sympherobius arizonicus* Banks	KX223392	KX085024	KX247667	SYMAR	USA: Texas: El Paso, S.L. Winterton, August 2001, light sheet
*Sympherobius gayi* Navás	KX223393	KX085025 *	KX247668	SYMCH	CHILE: Valparaiso Quillota, Campanas National Park, Palmas de Ocoa, -32.932, -71.078, 215 m, 2.x.2000, malaise trap, M. E. Irwin & E.I. Schlinger
*Sympherobius barberi* Banks	KX223394	KX085026 *	–	SYMBAR	USA:Texas: College. S.L.Winterton-JDO. viii.01. Det. JDOswald.
*Wesmaelius involutus* (Carpenter)	KX223395	KX085027 *	–	WESINV	USA: New Mexico: Cloudcroft, August 2001, S.L. Winterton & J.D. Oswald
*Wesmaelius subnebulosus* (Stephens) *Wesmaelius subnebulosus*	AY620149–	–KJ592492 *	––	WESSUB Wessub2 in Haring and Aspöck [5] BCZSMNEU168 in Morinière et al. [51]	Wessub2: FRANCE: Carcès BCZSMNEU168: GERMANY: Bavaria, Oberbayern, Freising, Allershausen
*Zachobiella lobata* New	KX223396	KX085028 *	–	ZALO	AUSTRALIA: Western Australia: Leeuwin-Naturaliste N.P., 16.xi.2008; -34.051, 115.018. S.L. Winterton & S.D. Gaimari.

Hemerobiidae and outgroup taxa used for DNA analysis in this study. We modified voucher codes to maintain a consistent labeling across our tree but the original voucher code is provided as well. Vouchers followed by an asterisk indicates incomplete fragments

### Morphological characters

The morphological matrix corresponds (with modifications) to that used by Oswald [3]. We revised the definition of some characters (Appendix 1) and excluded six characters (numbered 25, 30–34 in Oswald’s [3] original character list) that either pointed to the same statement of homology thus adding redundancy, or were subjective in interpretation and thus the identity of competing homologs was obscure. In the case of the modified characters, the original homology statement was maintained. We used 101 characters (92 phylogenetically informative). All character states were treated as unordered in all analyses. *Notherobius* was not originally included in the analysis by Oswald [3] as material was not available, but recently obtained material and DNA sequences included here enabled us to add the genus into the analysis. Missing characters states were scored as ‘?’.

### DNA extraction and gene sequencing

Genbank accession numbers, specimen voucher numbers and collection data are presented in Table 1. Adult specimens were placed into 95–100 % EtOH and stored at -80 °C. Genomic DNA was extracted from thoracic muscle tissue carried out using the DNeasy® kit (Qiagen, Maryland, USA) as per the manufacturer’s instructions except that specimens were incubated in the extraction buffer/proteinase-K mixture for 24 h. Three partial gene loci were amplified and sequenced, specifically chosen to represent a range of mutational rates thereby giving the best possibility for phylogenetically informative data across taxa sampled. Two mitochondrial genes were sequenced (*16S rDNA* and *cytochrome oxidase I* (COI)) along with a single nuclear gene, *CPSase region of carbamoyl-phosphate synthetase-aspartate transcarbamoylase-dihydroorotase* (CAD)). Primer sequences used to amplify and sequence the three gene regions are presented in Table 2. DNA amplifications using polymerase chain reaction (PCR) were performed using the following cycling parameters. A *ca*. 550 bp fragment of 16S rDNA (3′-end) was generated using a single primer pair originally from Simon et al. [15] with the following PCR protocol: initial denaturation 95 °C (3 min.); five cycles of 92 °C (15 s.), 48 °C (45 s.), 62 °C (2 min. 30 s); 29 cycles of 92 °C (15 s.), 52 °C (45 s.), 62 °C (2 min. 30 s.); final extension at 62 °C for 7 min. The 3′ end of COI DNA (ca. 500 bp) was amplified using primers modified after Simon et al. [15]: initial denaturation 94 °C (2 min.); 35 cycles of 94 °C (40 s.), 55 °C (50 s.), 72 °C (1 min.); final extension at 72 °C for 10 min. Fragment 1 of CAD [16] was generated using a touchdown PCR with the following conditions: initial denaturation at 94 °C (4 min.); five cycles of 94 °C (30 s.), 54 °C (30 s.) and 72 °C (90 s.); 37 cycles of 94 °C (30 s.), 51 °C (30 s.) and 72 °C (9y.); 72 °C (3 min.) for final extension.

**Table 2:** Primers used to amplify and sequence the three gene fragments used in this study

Fragment	Primer sequence	Source
16S	(LR-J-12887 F) CCGGTTTGAACTCAGATCATGT	[15]
	(SR-N-13398R) CRCYTGTTTAWCAAAAACAT	
COI	(TY-J-1460 F) TACAATCTATCGCCTAAACTTCAGCC	[15]
	(C1-N-2191R) CCCGGTAAAATTAAAATATAAACTTC	
	(C1-J-2195 F) TTGATTTTTTGGTCACCCTGAAGT	
	(TL2-N-3014R) TCCATTGCACTAATCTGCCATATTA	
CAD	(338 F) ATGAARTAYGGYAATCGTGGHCAYAA	[16]
	(680R) AANGCRTCNCGNACMACYTCRTAYTC	

Sequences were obtained using Applied Biosystems Big Dye Terminator V3.0 (Foster City, CA, USA). Sequences were gel fractionated and bases called on an ABI 3730TM DNA sequencer (PE Applied Biosystems). Sequencing electropherograms were edited and contigs assembled and proofed using SequencherTM 5.3 (GeneCodes Corp., Michigan, USA) and Geneious 7.1.7 [17].

### Sequence alignment and phylogenetic analyses

Alignment of all sequences was done manually, although CAD and COI were aligned with reference to translated amino acid sequences using Mesquite 3.02 [18]. All alignments were relatively straightforward, with few ambiguous regions present in the ribosomal sequence data and no introns in the protein coding genes (PCGs). Parsimony analyses on the morphological, molecular and combined datasets were conducted in TNT [19] using a heuristic search that included 500 replicates of random addition sequence, holding 10 trees per replication after tree bisection and reconnection (TBR) for branch swapping and 90 iterations of ratchet [20]. Gaps were read as missing data in the parsimony analyses. In all cases, branch support was assessed by Jackknife calculated from 1,000 pseudoreplicates of re-sampled data sets. Bayesian analyses were performed on the molecular and combined datasets using MrBayes 3.2.3 [21]. To assess the best fitting model and partitioning scheme of the data set before proceeding with the phylogenetic analysis, we used PartitionFinder (PF) [22] under the following settings: branchlenghts were set as unliked, the search was conducted under the greedy algorithm and the BIC (Bayesian information content) was used for model selection. The results from PF suggested three models in the GTR family as the best fitting models (GTR + I + γ, HKY + γ and TrN + γ) for three character sets (1: 16S, COI 1^st^, 2^nd^ positions, CAD 1^st^, 2^nd^ positions; 2: CAD 3^rd^ positions, 3: COI 3^rd^ positions). In MrBayes the *nst* command was set to *mixed* and *rate* to *gamma* which specify model averaging over the family of GTR models. For the morphological partition a gamma distribution was used with *coding* and *ratepr* commands set to variable. In all cases all the parameters in the model were unlinked. Each analysis consisted of four MCMC chains run simultaneously for 55 million generations. Trees were sampled every 500th generation and the burn-in fraction was set to 0.25 (25 %). Convergence was assessed using the standard deviation of split frequencies diagnostic given by MrBayes, set to stop the chain once a value of 0.01 was reached. A majority rule consensus tree was calculated with posterior probabilities (PP) for each node. Finally, unambiguous morphological changes were plotted over this topology using MacClade 4.06 [23].

### Estimation of divergence times

We conducted a divergence time analysis in PhyloBayes 3.3 [24] using the CAT - GTR model that incorporates infinite mixture models and hence is better able to accommodate for heterogeneity in substitution rates [25]. Divergence times were estimated using the molecular data on the topology obtained in the phylogenetic analysis of the total evidence matrix. Among the different sources of error associated with the estimation of divergence times, rate variation is considered significant [26]; but disagreement persists on whether or not rate variation is a heritable attribute [27, 28]. Therefore, in PhyloBayes we used two relaxed clock models that differ in their assumption regarding the heritability of substitution rates: an uncorrelated gamma multipliers model (UGAM) that assumes no heritability of substitution rates [29], and a log normal autocorrelated model (LN) [30] that assumes that the substitution rate at the descendent branch conforms to an underlying distribution (e.g., lognormal) of the rate at its ancestral branch and estimates it from there. More than 30 fossils of hemerobiids have been described [31], the vast majority of them from tertiary-aged deposits [32], although there are older Mesozoic fossils, these require re-examination and do not correspond to extant lineages. We chose the age of four fossils corresponding to members of extant genera, which allows us to calibrate specific clades and used them as calibrations of minimum age. The Miocene fossils *Megalomus caucasicus* Makarkin [33], and *Notiobiella thaumasta* Oswald [34] were set to a minimum age of 15 myr and 20 myr, respectively. Likewise, two Eocene fossils, *Sympherobius siriae* Jepson et al. [31], and *Wesmaelius mathewesi* Makarkin et al. [32] were set a minimum age of 45 myr and 51 myr respectively. The root (the split of *Psychopsis* from the remaining taxa) was constrained with a uniform prior of 200–230 Myr following Winterton et al. [6]. A birth-death speciation model was assumed on divergence times. Two chains were ran in PhyloBayes under each relaxed clock model (UGAM and LN) for 39,266 and 28,242 cycles respectively and the posterior chronogram was obtained after discarding the first 3,000 and 2,000 saved cycles respectively as burn-in.

## Results

### Phylogenetic analysis

The total sequence length after alignment was 2760 base pairs (bp), comprising 542 bp of 16S (229 variable sites), 876 bp of CAD (503 variable) and 1478 bp of COI (568 variable). A 49 bp long A-T rich fragment of 16S and an indel rich 88 bp length of CAD fragment were both unalignable with any confidence in homology and were excluded from the phylogenetic analysis. Exploratory phylogenetic analyses were carried out excluding third codon positions, but were discarded since in each instance it reduced tree resolution noticeably and violated monophyly of well-supported clades based on extraneous evidence (e.g., monophyly of Hemerobiidae) [35].

**Fig. 4:**
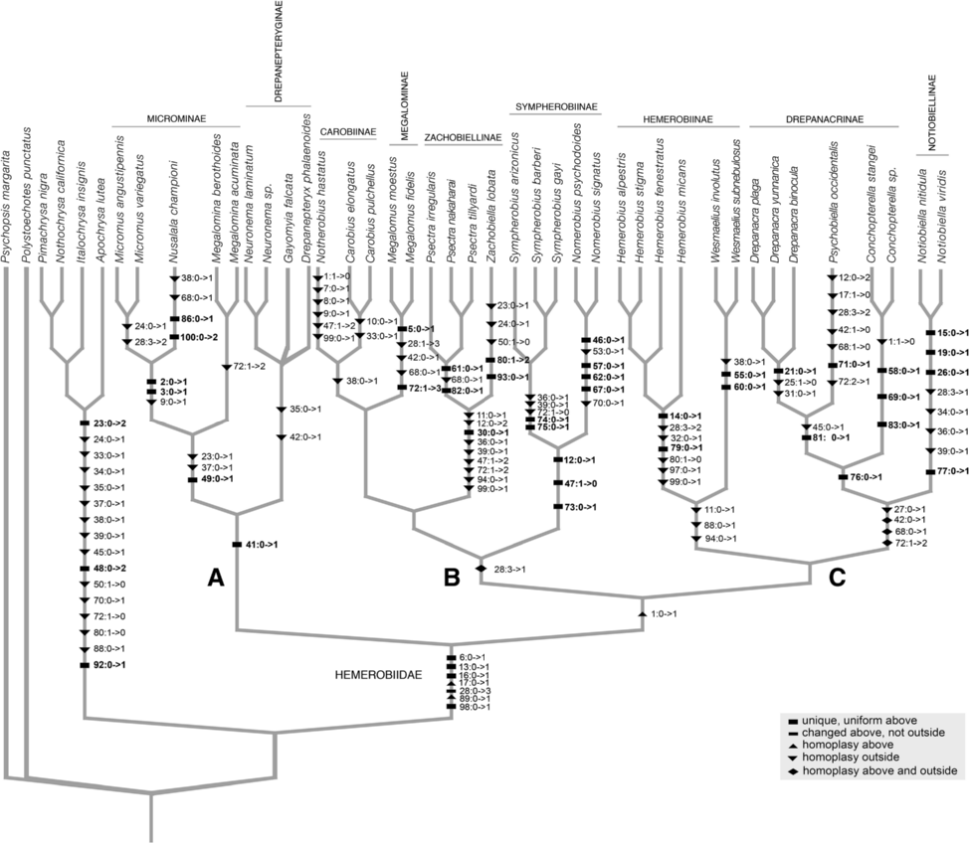
Character optimization of unambiguous morphological transformations on the topology obtained with the combined evidence in MrBayes

Separate analyses of either the morphological partition or each molecular marker (with parsimony or Bayesian inference) produced topologies with little resolution, suggesting that neither character system is capable of producing a robust hypothesis in isolation (Additional files 1, 2, and 3). By combining the morphological and molecular partitions we were able to obtain a well-resolved tree with relatively strong support under Bayesian inference; by contrast Parsimony produced a topology with low support and largely lacking resolution. In all Bayesian analyses, Hemerobiidae were monophyletic (posterior probability (PP) = 1.0) and sister to Chrysopidae; likewise we found all but two subfamilies (Notiobiellinae and Drepanacrinae) as monophyletic. Notiobiellinae was found polyphyletic, with *Notiobiella* as sister to Drepanacrinae, while the remaining Notiobiellinae genera (*Zachobiella* and *Psectra*) were recovered sister to Megalominae and Carobiinae. Drepanacrinae was rendered paraphyletic by the inclusion of Psychobiellinae (i.e., *Psychobiella*) with a high level of statistical support in all analyses. Figure 4 features the unambiguous morphological transformations plotted over the Bayesian topology obtained with the combined evidence. All monophyletic subfamilies (except for Drepanepteryginae) were supported by morphological characters transformations (Fig. 4). The monophyly of Hemerobiidae is supported by four unique synapomorphies and three homoplasious transformations.

The combined tree (Fig. 3) supports the existence of three major clades within Hemerobiidae (denoted as A, B and C), yet the relationships to each another are equivocal based on the available evidence. The three major clades recovered are each individually well supported (PP > 0.9) and comprise the following lineages: Clade A, contains the subfamilies Microminae and Drepanepteryginae, and is sister to the remaining Hemerobiidae. Clade B contains the subfamilies Carobiinae, Megalominae, Sympherobiinae and a new subfamily (Zachobiellinae) containing genera formerly placed in Notiobiellinae. Clade C contains *Notiobiella* as the sole genus in Notiobiellinae, as well as Hemerobiinae and Drepanacrinae (including the former Psychobiellinae).

### Age and divergence times

Figure 6 features the chronograms obtained by differing clock models. Node dating using the Lognormal auto correlated model (LN) (Fig. 6a) estimated the (mean) crown age of the major clades in Hemerobiidae as follows (with 95 % highest posterior density intervals [HPD] in parentheses): Hemerobiidae: 143.25 myr (170.48 – 116.46), Microminae: 118.82 myr (151.68 – 84.76), Drepanepteryginae: 125.17 myr (155.92 – 94.35), Notiobiellinae: 95.97 myr (129.46 – 63.50), Megalominae: 63.55 myr (101.41 – 31.51), Carobiinae: 61.38 myr (89.52 – 37.95), Sympherobiinae: 91.87 myr (124.28 – 64.43), Hemerobiinae: 112.60 myr (143.08 – 84.33), Drepanacrinae: 134.02 myr (161.98 – 106.62). The ages for these same nodes under the Uncorrelated Gamma Multipliers (UGAM) model were (Fig. 6b): mean crown age for Hemerobiidae: 163.78 myr (189.84 – 134.65), Microminae: 112.52 myr (158.73 – 62.49), Drepanepteryginae: 129.82 myr (167.52 – 82.13), Notiobiellinae: 107. 74 myr (139.84 – 74.49), Megalominae: 38.4 myr (75.77 – 18.29), Carobiinae: 52.45 myr (86.70 – 27.25), Sympherobiinae: 98.94 myr (133.18 – 67.34), Hemerobiinae: 103.51 myr (145.39 – 68.32), Drepanacrinae: 140.84 myr (172.92 – 103.64). Under both clock models the split between Hemerobiidae and Chrysopidae was estimated around the end of the Triassic and beginning of the Jurassic, with a mean age and HPD of 199.13 myr under LN (220.14 – 175.30) and 201.60 myr under UGAM (222 – 176.79).

## Discussion

During the last 100 years Hemerobiidae has undergone a variety of taxonomic arrangements that divided its current members at first in two separate families [36], subsequently into multiple subfamilies [37, 38], tribes [39] and more recently again as subfamilies [3]. Although the tree we obtained agrees largely with Oswald’s [3] classification, with most subfamilies recovered as monophyletic, it differs from Oswald’s in the arrangement of the relationships among various subfamily groups. Notably, with hemerobiids diverging as three main clades (Fig. 3) versus Oswald’s [3, 10, 11] exceptionally imbalanced (i.e., laddered) topology (inset in Fig. 3), and in the placement of Carobiinae as a relatively derived group rather than sister to the rest of the family (exclusive of Adelphohemerobiinae). We recovered seven of the nine previously defined Hemerobiidae subfamilies sampled here as monophyletic groups with 100 % posterior probability support. Drepanepteryginae was found as monophyletic, although with low posterior support. All the genera represented in our study by more than one species were found to be monophyletic and recovered with strong posterior support, yet relationships among some genera were more difficult to recover with confidence, again particularly within Drepanepteryginae.

### Origin and monophyly of Hemerobiidae

Hemerobiidae are a well-supported monophyletic family of lacewings based on a series of morphological and molecular characters (Fig. 4). Apomorphies of the family (based on Oswald’s characters used here) include, the presence of peniciliform sensillae on the galea (6: 1), clypeus with paired dorsocentral and ventrolateral setae (13:1; 16:1), and multiple oblique radial veins originating on R1 in the forewing (28:3). The last character has been used to define the family, as the plesiomorphic state is a single radial vein originating on R1 (28:0) and is found in all other extant Neuropterida. The number of radial veins (also known as ORBs or radial sectors) varies among genera of Hemerobiidae (Figs. 2 and 7), from two veins in subfamilies Sympherobiinae, Zachobiellinae subfam. nov., Carobiinae (all clade B) and Notiobiellinae (clade C), three radial veins in some Microminae (e.g., *Micromus*), Hemerobiinae (e.g., *Hemerobius*) and Drepanacrinae (e.g., *Psychobiella*) to more than four radial veins in some members of Microminae (e.g., *Nusalala*), Hemerobiinae (e.g., *Wesmaelius*), Depranacrinae (e.g., *Conchopterella*) and in all members of Drepanepteryginae.

Divergence time estimates for Hemerobiidae based on the total evidence tree obtained here suggest that the family diverged from Chrysopidae during the Late Triassic or Early Jurassic, depending on the clock model used (Fig. 6a: UGAM, 201 Ma; Fig. 6b: LN, 199 MA). This is slightly older than that proposed by Winterton et al. [6], but still falls within their HPD range. The difference in divergence time estimates recovered here under the two clock models, while distinct, is still within the ranges of the HPD, particularly for older nodes and is similar to the pattern reported by Sharma and Giribet [40] in Opiliones, a study that also explored the effect of these two clock models. As mentioned previously, the principal difference between the two models is in their assumptions of heritability of substitution rates [41], the LN allowing for substitution rates to be inherited (i.e., autocorrelated), while UGAM does not consider the rate a heritable attribute, and thus rates at different depths of the tree are considered independent. As in Sharma and Giribet [40], we found that age estimates for unbound nodes (lacking actual or proximal minimal age constraints) were variable based on the choice of clock model. This is particularly the case for older (Jurassic aged) nodes lacking minimum age constraints. Here, we used only Cretaceous and Palaeogene aged fossils that were confidently associated with crown lineages (i.e., extant genera) as minimum age constraints. Two Mesozoic-aged hemerobiids are known, *Promegalomus anomalus* Panvolov in Dolin (Late Jurassic) and *Cretohemerobius disjunctus* Ponomarenko (Early Cretaceous), but both are stem fossils and not assignable to any crown lineage. Consequently, while they do provide a minimum age for the family Hemerobiidae, they do not provide enough information to place a minimum age on any particular subfamilial lineage. Regardless of the clock model used, both analyses resulted in wide confidence intervals around the mean. Estimates of divergence times are improved by having as many calibrations as possible around nodes of interest; when trying to elucidate the evolutionary time table for a family such Hemerobiidae, this means having (ideally) calibrations spread across the tree. Therefore the wide confidence interval are most likely a reflection of our shallow calibrations being unsuitable to accommodate the variation found at deeper parts of the tree.

Based on the age of the oldest hemerobiid fossil and the presence of genera with species in both Old World and New World, Oswald [3] suggested a Mesozoic origin for the family. Our analyses of divergence times support this hypothesis, further indicating an Early Mesozoic origin. Likewise, our estimation of the crown age of Hemerobiidae and that of its split from Chrysopidae fall within the confidence intervals of previous estimations by Winterton et al., [6] done with a limited taxonomic sample. It should be noted that this result was obtained despite the root of Hemerobiidae being left unconstrained. As they diverged early in the Mesozoic, the ancestral brown lacewings were probably widespread in Pangaea. Furthermore, we found Australian and Pacific genera included in Microminae, Carobiinae Drepanacrinae, Psychobiellinae and Notiobiellinae represented throughout the tree and not forming distinctive clades. According to our divergence time analyses, by the Cretaceous splitting of Gondwana, Australasian and southern South American clades were already differentiated, suggesting that their present distributions are due to much older vicariance. A similar historical biogeographic pattern also found in Sialidae (Megaloptera) [42]. Finally, it is noteworthy that the two Chilean genera, i.e., *Nomerobius* (Sympherobiinae) and *Conchopterella* (Drepanacrinae), were estimated to have diverged largely contemporaneously under the UGAM model (91.8 myr and 91.4 myr, respectively). This suggests that the initial separation between southern South America + Antarctica and Australia during 90 myr [43] might account for the formation of these groups due to geographic vicariance.

### Clade A: Drepanepteryginae and Microminae

**Fig. 5:**
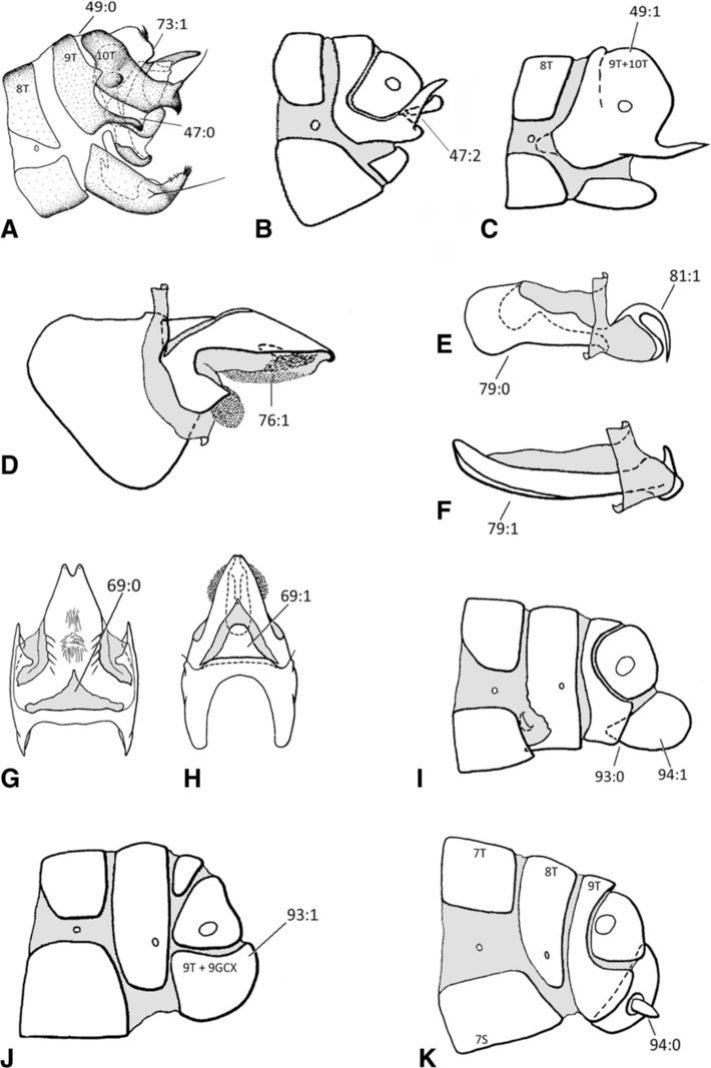
Male and female genitalia characters supporting relationships among Hemerobiid genera. **a** Male genitalia of *Nomerobius* in lateral view, **b** Male genitalia of *Psectra* in lateral view, **c** Male genitalia of *Micromus* in lateral view, **d** Gonarcus of *Conchopterella* in lateral view*,* **e** Parameres in *Psychobiella* in lateral view, **f** Parameres in *Hemerobius* in lateral view, **g** Gonarcus of *Drepanacra* in dorsal view, **h** Gonarcus of *Conchopterella* in dorsal view, **i** Female genitalia of *Hemerobius* in lateral view, **j** Female genitalia of *Zachobiella* in lateral view, **k** Female genitalia of *Carobius* in lateral view (Drawings from [3–52]

Drepanepteryginae and Microminae (Clade A) are strongly supported here as sister groups (PP: 0.99). This was also found by Oswald [3] although the placement of these two subfamilies relative to the rest of Hemerobiidae is opposite to the highly derived position of this clade in Oswald’s [3] phylogeny. Here, a few nucleotide substitutions (5) of 16S and COI and one unambiguous morphological change supports this sister group relationship (the presence of an intercubital crossvein 1cua-cup in the forewing (41:1)). Based on the divergence time estimation, Drepanepteryginae and Microminae diverged from the rest of the family during the Late Jurassic -Early Cretaceous (UGAM: 163/ LN: 143 Ma). Drepanepteryginae is represented by three genera, *Drepanepteryx* (Palaearctic, Oriental), *Neuronema* (Oriental) and *Gayomyia* (Neotropical). Relationships among these genera are equivocal in this analysis, but Oswald [3] recovered *Drepanepteryx* as sister to *Gayomyia* based on three homoplasious transformations. All members in this subfamily are large, distinctive hemerobiids characterized by their broad, often falcate wings usually with numerous radial veins and broad humeral costal area. Microminae is represented by *Micromus* (cosmopolitan), *Megalomina* (Australasia), *Nusalala* (Neotropical) and *Nois* (New Caledonia), with all but *Nois* sampled here. The subfamily is well supported here as defined by Oswald [3] and is characterized by a single unambiguous synapomorphy, male abdominal tergites 9 and 10 fused (49:1) (Fig. 5c) and two homoplasious transformations (23:1 and 37:1). Differing slightly from Oswald [3], we recovered *Nusalala* as sister to *Micromus* rather than *Megalomina,* all with high levels of support.

### Clade B: Sympherobiinae, Zachobiellinae subfam. nov., Megalominae and Carobiinae

Clade B diverged from Clade C during the Late Jurassic to Early Cretaceous and comprises four subfamilies that are united by a single homoplasious character transformation, the reduction of forewing radial veins to two (28:1). This is a not a homogenous feature in the clade with multiplication of FW radial veins occurring once again in Megalominae (28:3). We found the previously enigmatic genus *Notherobius* New to be sister to *Carobius* Banks and thus part of Carobiinae. This relationship was obtained in the DNA-based tree and in the tree with combined evidence in both cases with high PP values. Oswald [3] discussed the potential phylogenetic affinities of *Notherobius* to Sympherobiinae (based on the presence of two prestigmal radial veins and the presence of styli on the female 9^th^ gonocoxites) although he rejected this hypothesis arguing the lack of synapomorphic features and consequently did not provide a definitive position for the genus. The presence of a mediocubital crossvein 3 m-cu also supports (char. 38:1) this relationship (present also in Chrysopidae). This represents a novel finding for a genus whose phylogenetic origins had remained obscure for almost 30 years [44]. Indeed, New [44] remarked on the general similarity in appearance between *Notherobius* and *Carobius* and the sister group relationship between the two genera is now unambiguous.

The sister-group relationship between Megalominae (including *Megalomus*) and Carobinae is relatively weakly supported, with no synapomorphies for the clade identified. Oswald [3] instead placed Megalominae as sister to Drepanepteryginae + Microminae, although again it was weakly supported with few non-homoplasious character changes. The placement of this subfamily remains ambiguous and remains an area requiring further study.

Our analysis corroborates the close relationship of *Zachobiella* and *Psectra* with strong statistical support, as found by Oswald [3], although in that case including also the highly autapomorphic genus *Anapsectra*. Defined here as a new subfamily Zachobiellinae, this clade is one of the most distinctive and well supported clades (Figs. 3 and 4) in the family. Eight homoplasious and one synapomorphic character state changes support its monophyly. The single unique feature of this clade is the secondary absence of wing intraradial crossvein 4ir1 (30:1). Our results similarly corroborate the monophyly of Sympherobiinae (including *Nomerobius*, *Sympherobius* and *Neosympherobius*). The sister relationship between the genera included here (*Nomerobius* and *Sympherobius*) is supported by three unique morphological transformations: a well developed and rounded distal convexity of the orad margin of the right mandible (char. 12:1), the posteroventral angle of the 9^th^ tergite as a narrow membrane-margined lobe (char. 47:0) (Fig. 5a), and the presence of a pseudomediuncus (char. 73:1) (Fig. 5a).

### Clade C: Hemerobiinae, Notiobiellinae and Drepanacrinae

**Fig. 6:**
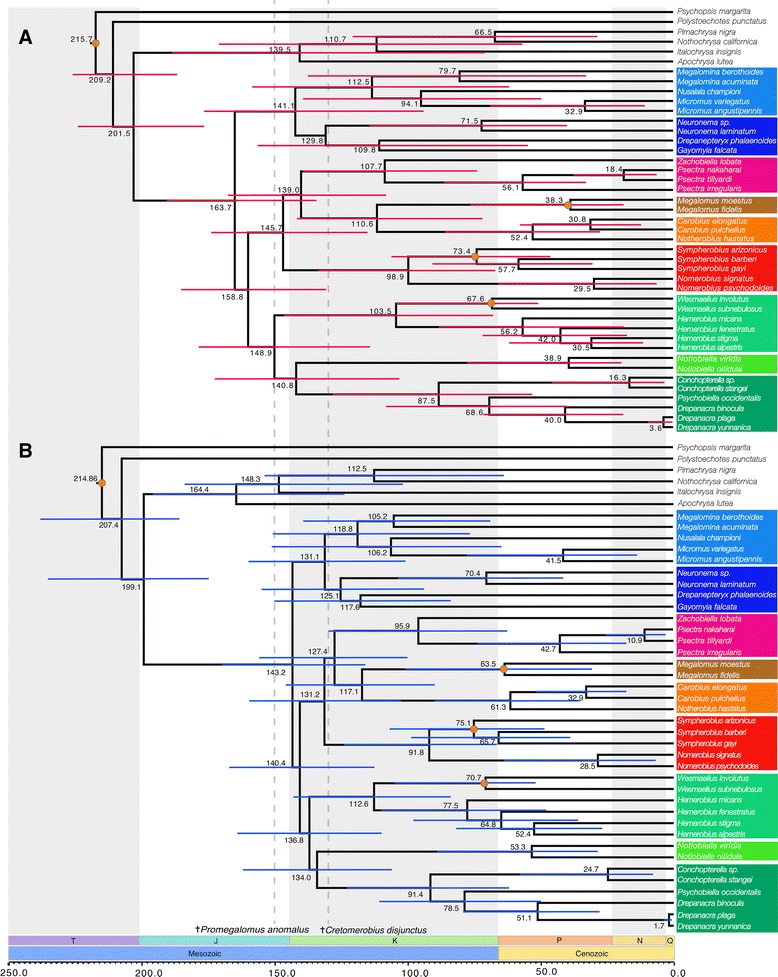
Chronograms obtained from the divergence times analyses in PhyloBayes under two relaxed molecular clocks. **a** = uncorrelated gamma multipliers model (UGAM), **b** = Log normal autocorrelated model (LN). Orange circle denote minimum age constraints for a node based on ages of crown group fossils definitively placed in that genus. Dashed vertical lines represent ages of both stem group fossils definitively paced in Hemerobiidae but not associated with any crown group

The three families comprising Clade C (i.e., Hemerobiinae*,* Notiobiellinae (sensu stricto) and Drepanacrinae (inclusive of Psychobiellinae)) are well supported based on molecular data, but lack any morphological synapomorphies to support the clade. Oswald [3, 10] failed to recover these families in a clade, although they were placed relatively close to each other in his proposed phylogenies. In our analyses, Hemerobiinae is sister to Drepanacrinae + *Notiobiella* (Notiobiellinae *s.s.*). Drepanacrinae (represented here by *Drepanacra* and *Conchopterella*) was rendered paraphyletic by the inclusion of the Australian genus *Psychobiella* (Psychobiellinae)*.* This result was obtained with the molecular and combined evidence with 100 % posterior probability values in both cases. Consequently, we consider Psychobiellinae syn. nov. (containing only *Psychobiella*) as a synonym of Drepanacrinae. Drepanacrinae can be defined by the presence of a scabriculous region in the gonosaccal membrane (below mediuncus) (char. 76:1) (Fig. 5c), which is here a unique and universal character transformation (according to Oswald [3] this character reverses in the genus *Austromegalomus*, which was not included in this study). The sister relationship between *Psychobiella* and *Drepanacra* was found by the DNA evidence alone and by the combined evidence, in both cases with high statistical support values. Figure 6d illustrates a unique morphological transformation of this clade: the presence of dorsal subapical spinose processes of the parameres (char. 81: 1). Likewise the monophyly of *Conchopterella* is supported by multiple character transformations (Fig. 4); one of the more distinctive being the presence of a gonofenestral plate (char. 69:1), (Fig. 5h) (cf*.* alternative character state in Fig. 5g).

*Notiobiella* Banks was consistently found sister to Drepanacrinae once the morphological and molecular evidence were combined, although with modest posterior support values. It should be noted though, that Notiobiellinae (sensu *lato*) as defined by Oswald [3], was recognized solely by losses of wings crossveins and that Oswald himself questioned the grouping of *Notiobiella* with the clade formed by *Psectra*, *Anapsectra* and *Zachobiella.* Likewise, Nakahara [38] suggested the separation of *Notiobiella* from all others hemerobiids into its own subfamily, mainly based on the presence of the phallolingua, which remains an autopomorphy of *Notiobiella* (char. 77:1).

Although lacking representatives of *Nesobiella, Biramus* and *Hemerobiella*, our study corroborates the monophyly of Hemerobiinae based on multiple representatives of *Hemerobius* and *Wesmaelius*. This clade was supported with 100 % posterior probability values in both the molecular and the combined evidence trees. Figure 5i illustrates one of the morphological changes supporting the monophyly of the subfamily: the loss of stylus on 9^th^ gonocoxite (char. 94:1, Fig. 5i vs. Fig. 5k), which is also absent in *Psectra*, *Zachobiella*, *Drepanepteryx*, *Nusalala*, *Micromus* and *Megalomina*. Two other homoplasious changes support this clade: the presence of a supragonopontal setal group (char. 88:1) and proximal convexity of orad margin of right mandible, prominently convex and strongly angulate (char.11:1). The monophyly of *Wesmaelius* and *Hemerobius* is supported by multiple apomorphies (Figs. 4), including one of the synapomorphies of *Hemerobius*, parameres deeply (entirely or nearly entirely) divided, composed of a pair of adjacent, narrow sclerotized straps (char. 79:1 Fig. 5f).

The sister relationship between clade B and C was only recovered with the combined evidence analysis and was not recovered in trees sourced solely from either molecular data or the morphology data (Additional files 1 and 2). Indeed, in trees sourced from either dataset this particular node was equivocal. With the combined evidence, several nucleotide substitutions were found at this node but only one unambiguous, homoplasious morphological transformation (char. 1:1): the presence of a well developed temporal costa, (reversed in *Notherobius* and *Conchopterella)*. The posterior probability value for this node varied from 0.5 to 0.80 under different analytical settings.

### Adelphohemerobiinae and the radial vein proliferation hypothesis

**Fig. 7:**
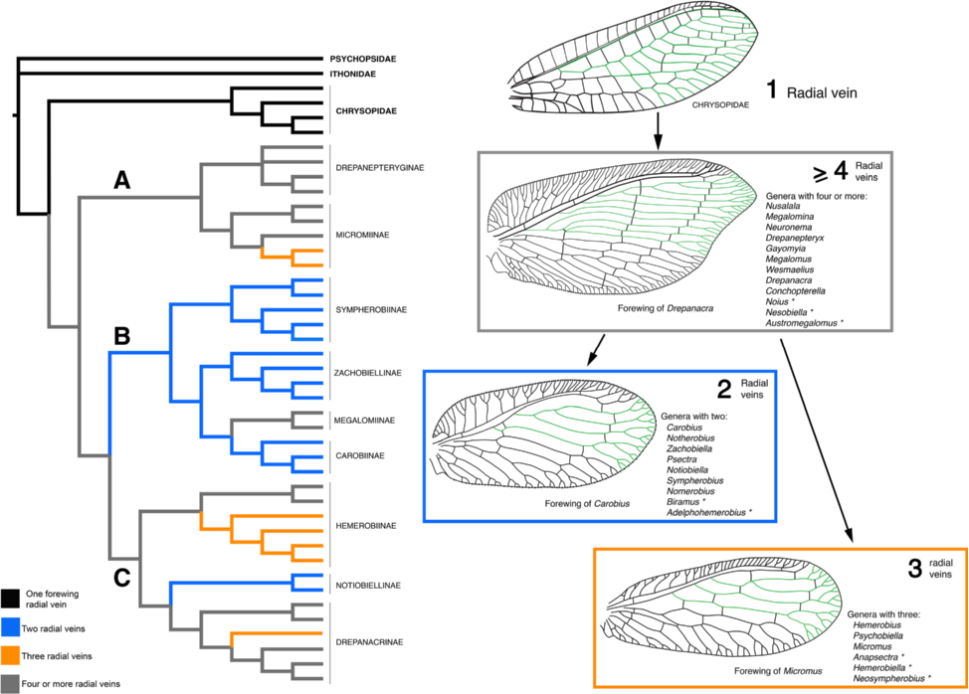
Parsimonious unambiguous optimization of the number of forewing radial veins (also called ORBs) (character 28) on the combined evidence phylogeny resulting from the Bayesian analysis. Wing figures of radial veins arising from R1 are highlighted in green from the stem Chrysopidae outgroup and in examples of derived Hemerobiidae lineages (*Drepanacra*, *Carobius* and *Micromus*). Colors of boxes and particular tree lineages correspond with the number of forewing radial vein branches

In light of the current available evidence based on the combination of morphology and DNA sequences, our estimate of Hemerobiidae phylogeny calls into question the proposed scenario by Oswald [3, 10, 11] regarding the evolution of the multiple forewing radial veins, and to an extent too, the relevance of this character in the diagnosis of various subfamilies. As pointed out by Hennig [45] the recognition of derived venation characters is important for evaluating fossils. Although a single radial vein originating on R1 is considered plesiomorphic within Neuroptera [10, 11], a progressive increase in radial veins originating on R1 within Hemerobiidae [10] is not supported by our topology. In fact a parsimonious character reconstruction favors the presence of multiple forewing radial veins as the plesiomorphic condition in Hemerobiidae (Fig. 7) with multiple reductions in number occurring in separate lineages, a scenario proposed by Tillyard [46] and supported by subsequent authors (e.g., Nakahara [38]). The only instance of an increase in the number of radial veins occurs in clade B (i.e., Megalominae). Within this clade there is a character transformation from state 1 (two radial veins) to state 3 (four or more). In clades A and C, four or more radial veins is the typical plesiomorphic condition with transformations to fewer veins occurring four times independently. In clade A this occurs within Microminae and in clade C in Notiobiellinae, Drepanacrinae and within Hemerobiinae. Finally, although genera with a similar number of radial veins do largely fall within the same clades (albeit with some variation in number within a genus), none of the three clades can be diagnosed by one of the three character states. Tillyard [46] and Nakahara [38] proposed that genera with multiple radial veins, such as *Drepanepteryx* and allied genera represented some of the most ‘generalized forms’, with a gradual reduction in the number of veins in more derived genera such as *Psychobiella* and *Notiobiella*, with *Carobius* represented as side-lineage and not as sister to the rest of the family. The basis for their argument was on the overall structure of the male genitalia rather than on the somewhat variable nature of the wing venation.

Given that in our topology the sister relationship among clades B and C was weakly supported (the clades otherwise had all PP >0.90), and that alternative topologies could produce a different character optimization regarding the transformation in the number of radial veins. We conducted a character optimization under the two alternative scenarios: in the first one Clade C is sister to the rest of Hemerobiids (thus clades A and B are sister clades), in the second one, Clade B is sister to the rest of the ingroup (thus clades A and C as sister clades). Our hypothesis of multiple reductions of radial veins is supported also under the first scenario, and under the second scenario the ancestral character reconstruction would be equivocal. Thus, although the sister relationship of clades B and C was not decisively supported, a scenario where multiple reductions in the number of radial veins has occurred is not contingent upon this relationship, since having 4 or more radial veins is the ancestral character reconstruction at the root of Hemerobiidae in 2 of the 3 possible alternative topologies. In other words, under any reconstruction of relationship of clades A, B and C, no scenario supports the hypothesis of progressive increase in radial veins throughout the family.

A tenth subfamily, Adelphohemerobiinae, was not included in this analysis as it is known only from a single specimen of *Adelphohemerobius enigmaramus* Oswald [11]. According to Oswald [11] this taxon represents the putative sister to all other Hemerobiidae. This argument is based mainly upon the interpretation of a single forewing venation character in this specimen, specifically the identity of a particular vein as either a radial vein (=oblique radial branch) or as a vein internode (= crossvein). The evidence is weak either way and Oswald [11] preferentially interpreted this vein as a crossvein and thus presented a hypothesis of only a single radial vein arising from R1. As the only known specimen of *A. enigmaramus* is a female, this was done in the absence of male genitalic features that could have potentially confirmed or contradicted his hypothesis; the female genitalia, while unusual, are not necessarily considered to exhibit typical plesiomorphic characteristics. Oswald [11] lists various characters exhibited by *A. enigmaramus* in support of this ‘adelphotaxon’ hypothesis, but most represent plesiomorphies (i.e., absence of ‘scrobe’ on the basal maxillary palpomere; presence of subanale and 9^th^ gonocoxite stylus), autapomorphies (i.e., slit like insemination canal), or their homology is subject to interpretation (i.e., number of radial veins and distal subcostal crossvein position). Moreover, as Oswald [11] states, *A. enigmaramus* is a very typical hemerobiid. In contrast to Oswald, we interpret the crossvein 2ir1 of Oswald [11] as a second radial vein (albeit somewhat aberrant), and therefore we consider the genus more likely placed in the subfamily Sympherobiinae close to the genus *Neosympherobius*. Further evidence is required of this enigmatic taxon to place it with more certainty within Hemerobiidae, including addition of DNA sequence data and discovery of the male.

### Taxonomy

Zachobiellinae subfam. nov.

Type genus. *Zachobiella* Banks, 1920: 335.

Diagnosis. Small to medium size hemerobiids. Forewing intraradial crossvein 4ir1 absent; forewing intramedial crossvein 4im and mediocubital crossvein 4 m-cu absent; modification of the posteroventral angle of the 9^th^ tergite from a broad membrane-marginated lobe to an elongated subectoproctal lobe (usually) with a free distal process (state 2, Fig. 5b); labial palpomeres simple, bisubsegmentation lost; convexities of mandibles strongly angulate; apex of mediuncus emarginate; female 9^th^ gonocoxite lacking styli (Fig. 5j); female gonapophyses posteriores and subgenitale absent.

Included genera. *Anapsectra* Tjeder (Afrotropical), *Psectra* Hagen (Fig. 1d) (Afrotropical, Palaearctic, Oriental, Australasia, Oceania), *Zachobiella* Banks (Fig. 1b) (Australasia, Oriental).

Comments. The absence of the intraradial crossvein 4ir1 is an apomorphy for this clade but, according to Oswald [3] this crossvein is also absent in *Neosympherobius*, which was not included in this phylogenetic analysis. Oswald [3] noted when defining Notiobiellinae *s. l.,* that the subfamily was supported by three homoplasious characters involving losses of forewing crossveins and casting doubt on the robustness of the grouping of *Notiobiella* with *Psectra*, *Anapsectra* and *Zachobiella*. In contrast, the clade grouping the latter three genera was supported by multiple homoplasious characters and one apomorphic character change (Fig. 4). Our results corroborate this, placing *Notiobiella* (and Notiobiellinae *s.s.*) instead as sister to Drepanacrinae (inclusive of Psychobiellinae).

## Conclusions

The phylogenetic systematics of Neuroptera is steadily catching up to other holometabolous orders to produce family level phylogenetic hypotheses based on combined evidence [42, 47–49]. Adding to this endeavor, we present here the first total evidence phylogeny and divergence times estimation of intrafamilial relationships of the lacewing family Hemerobiidae. Our study shows that parts of the previous taxonomic arrangement based on morphology that had remained unaltered for the last 12 years are also supported by DNA evidence. Yet some aspects of Hemerobiidae phylogeny and classification are very different in our revised estimate of phylogeny for the family, including the definition of Notiobiellinae, erection of Zachobiellinae, synonymy of Psychobiellinae with Drepanacrinae, and the position of *Carobius*. Rather than a laddered set of relationships, we found the family is composed of three main lineages. Certain taxa not available for DNA sequencing should be the focus of future studies, especially *Adelphohemerobius*, which is particularly important to test our inferences regarding the evolution of wing venation in Hemerobiidae.

## Additional files

Additional file 1: Topology obtained with the molecular data alone under Parsimony. (PDF 342 kb) Additional file 2: Topology obtained with the molecular data alone under Bayesian Inference. (PDF 363 KB) Additional file 3: Parsimony topology obtained with the morphological data alone. (PDF 342 kb) Additional file 4: Morphological character list for Hemerobiidae (Neuroptera). (DOCX 21.8 kb) Additional file 5: Nexus file with instructions for Mr. Bayes. (ZIP 18.1 kb)

## Abbreviations

Bp: Base pairs
CAD: CPSase region of carbamoyl-phosphate synthetase-aspartate transcarbamoylase-dihydroorotase
COI: Cytochrome oxidase I
HPD: Highest posterior density
LN: Log normal autocorrelated model
MY: Million years
ORB: Oblique radial branches
PCG: Protein encoding genes
PP: Posterior probabilities
TBR: Tree bisection and reconnection
UGAM: Uncorrelated gamma multipliers model
