# Supplementary material for: The phylogeny of brown lacewings (Neuroptera: Hemerobiidae) reveals multiple reductions in wing venation
Source: BMC Evol Biol. 2016 Sep 20;16:192. doi: 10.1186/s12862-016-0746-5 (PMC5029026; doi:10.1186/s12862-016-0746-5)

Supplemental material 1. Majority rule consensus from a Bayesian inference analysis in Mr. Bayes of the molecular evidence alone. Branch support represent posterior probability values.

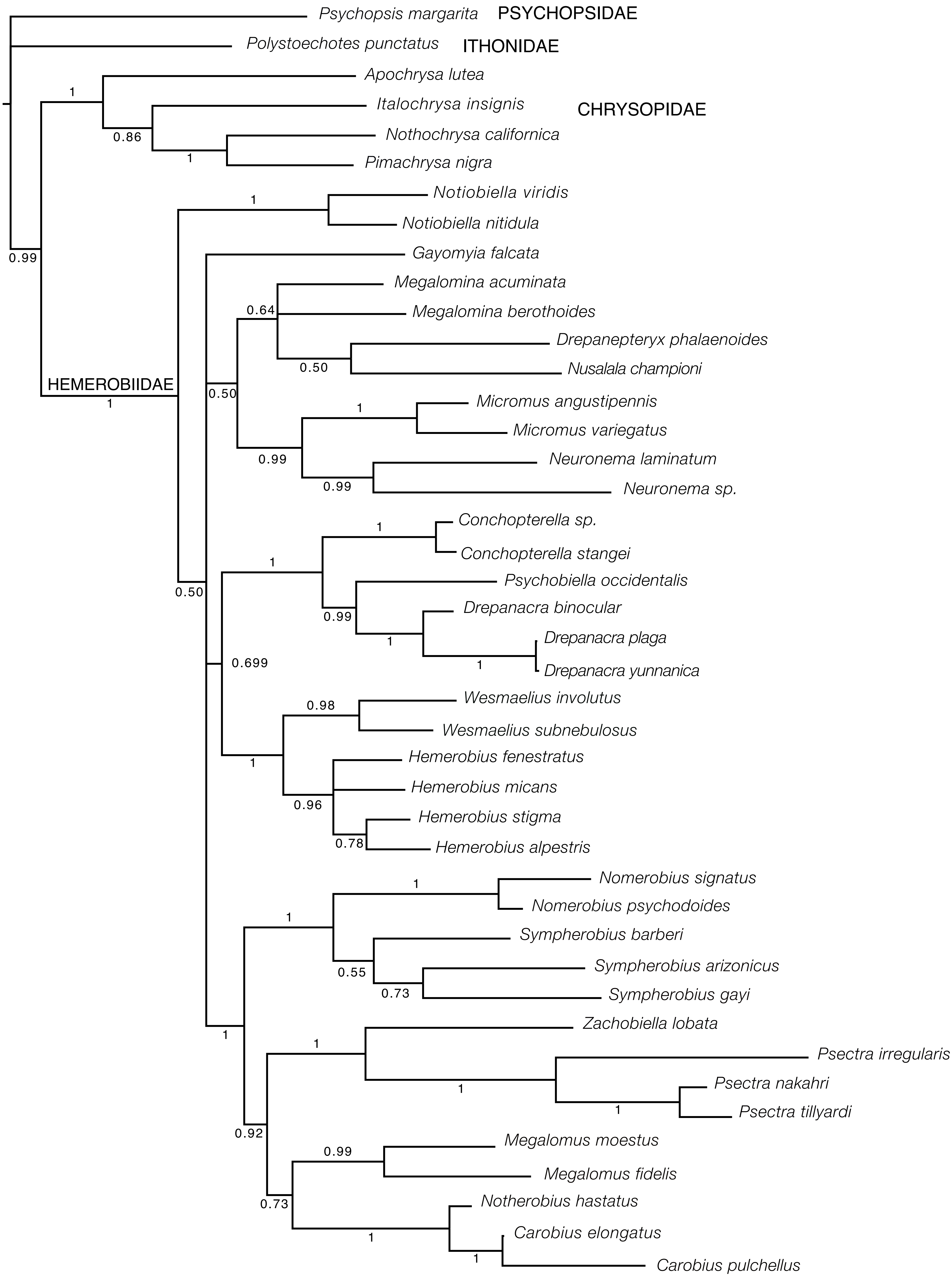

Supplement: Additional file 1: — Topology obtained with the molecular data alone under Parsimony. (PDF 342 kb) [file 12862_2016_746_MOESM1_ESM.pdf]
