# Supplementary material for: The phylogeny of brown lacewings (Neuroptera: Hemerobiidae) reveals multiple reductions in wing venation
Source: BMC Evol Biol. 2016 Sep 20;16:192. doi: 10.1186/s12862-016-0746-5 (PMC5029026; doi:10.1186/s12862-016-0746-5)

Supplemental material 2. Strict consensus of two most parsimonious trees obtained with equal weights Parsimony in TNT. Branch support corresponds to a 1000 replications of Jackknife.

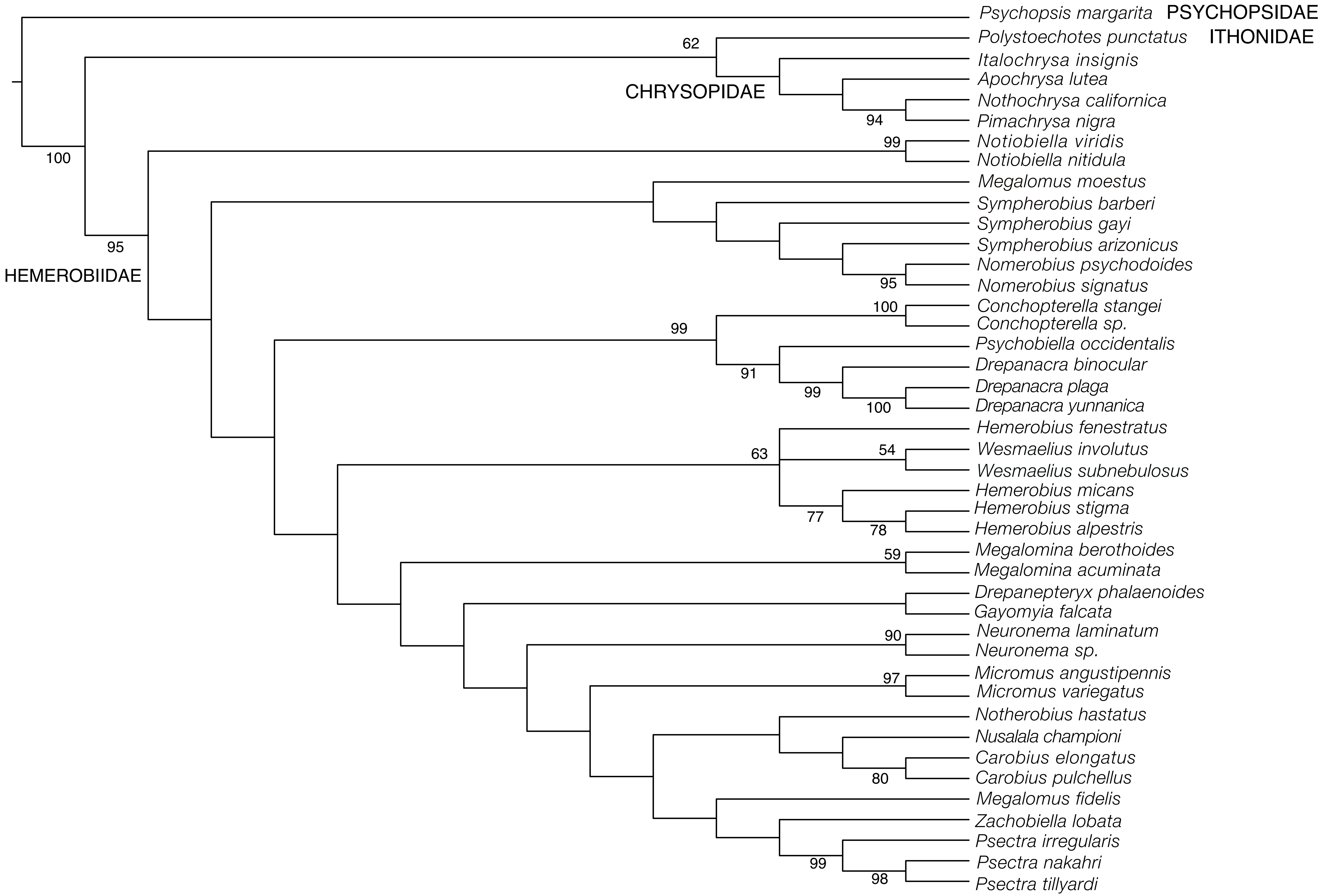

Supplement: Additional file 2: — Topology obtained with the molecular data alone under Bayesian Inference. (PDF 363 KB) [file 12862_2016_746_MOESM2_ESM.pdf]
