# Supplementary material for: The phylogeny of brown lacewings (Neuroptera: Hemerobiidae) reveals multiple reductions in wing venation
Source: BMC Evol Biol. 2016 Sep 20;16:192. doi: 10.1186/s12862-016-0746-5 (PMC5029026; doi:10.1186/s12862-016-0746-5)
Supplement: Additional file 4: — Morphological character list for Hemerobiidae (Neuroptera). (DOCX 21.8 kb) [file 12862_2016_746_MOESM4_ESM.docx]

**Additional File 4**. Morphological character list for Hemerobiidae (Neuroptera), modified from Oswald [3]. Note: Characters 78–83 mention the parabaculum, we have maintained Oswald’s original name in the character list, the reader should be aware this structure is usually called parameres by other authors [50].

1. Temporal costa: (0) absent or poorly developed; (1) well developed.

Note: this character was coded as (1) by Oswald for *Hemerobius*, *Psectra*, *and Zachobiella*, although he states that it is polymorphic.

2. Transtorular costa: (0) absent or poorly developed; (1) well developed.

Note: this character was coded as (1) by Oswald for *Micromus*, although he states that it is polymorphic.

3. Midfrontal costa: (0) absent or weakly developed; (1) well developed.

Note: this character was coded as (1) by Oswald for *Micromus*, although he states that it is polymorphic

4. Frontoclypeal costa: (0) entirely absent, or present only as a pair of spurs arising from the clypeogenal costae near the anterior tentorial pits, spurs not united medially; (1) present, a strong, transversely continuous, costa across the frontoclypeus below the anterior tentorial pits.

Note: this character was coded as (0) by Oswald for *Micromus*, although he states that it is polymorphic.

5. Columns of transverse strigae on epipharyngeal surface of labrum: (0) absent; (1) two columns present.

6. Peniciliform sensilla of galea: (0) absent; (1) present.

7. 5^th^ palpomere of maxillary palpus: (0) not subsegmented; (1) composed of two subsegments.

8. 3^rd^ palpomere of labial palpus: (0) not subsegmented; (1) composed of two subsegments.

9. Dorsal (cervical) margin of postmentum: (0) transverse or irregularly parabolic and poorly defined; (1) regularly parabolic and well defined.

Note: this character was coded as (1) by Oswald for *Nusalala*, although he states that it is polymorphic.

10. [Modified] Orad cavity of right mandible: (0) absent; (1) present.

Note: reduced to only two character states.

11. Proximal convexity of orad margin of right mandible: (0) prominently convex, rounded; (1) prominently convex, strongly angulate.

12. Distal convexity of orad margin of right mandible: (0) absent, (1) well developed, rounded; (2) well developed, strongly angulate.

13. Paired dorsolateral setae of clypeus: (0) absent; (1) present.

14. Sagittal seta of clypeus: (0) absent or short; (1) present and long.

15. Paired mesolateral setae of clypeus: (0) absent or short; (1) present and long.

Note: this character was coded as (0) by Oswald for *Neuronema*, although he states that it is polymorphic.

16. Paired ventrolateral setae of clypeus: (0) absent; (1) present.

17. Paired ventromedial setae of clypeus: (0) absent; (1) present.

18. Sagittal process of anterior tentorial bridge: (0) present; (1) absent.

19. Mesal lobes of anterior tentorial arms: (0) widely separated; (1) very closely adjacent or overlapping.

20. Setal foveae of cranium: (0) absent or few (<10) and inconspicuous; (1) numerous (>10) and prominent.

21. Field of punctulae on posterior margin of gena: (0) absent; (1) present.

22. Bilobed cavity of male frons: (0) absent; (1) present.

23. Trichosors of anterior wing margin: (0) present proximally and distally; (1) lost proximally, present distally; (2) lost proximally and distally.

24. [Modified] Humeral veinlet: (0) proximal humeral trace recurrent (1) simple.

Note: redefined and reduced to only two character states. Taxa that were coded as 2 were coded as 1. *Noius* and *Zachobiella* were recoded as (0).

25. [Modified] Crossveins [not veinlets] of coastal space: (0) regularly positioned, forming a distinct gradate series in costal space; (1) absent or few in number; (2) irregular in position, forming, with costal veinlets, a reticulate network in costal space at base of wing.

Note: redefined, *Drepanacra* and *Drepanepteryx* recoded as (0).

26. Prestigmal width of subcostal space: (0) narrow to broad, but > width of adjacent Sc throughout; (1) very narrow, ≤ width of adjacent Sc throughout.

27.Subcostal crossvein 2sc-r: (0) absent; (1) present.

Note: this character was coded as (1) by Oswald for *Notiobiella* and *Zachobiella*, although he states that it is polymorphic.

28. [Modified] Radial vein configuration: (0) 1 ORB, the true radial sector; (1) 2 ORB’s, (2) 3 ORB’s, (3) 4 + ORB’s, all genera characterized by 4 or more radial sectors are placed here.

29. Pre-3ir1 intraradial crossveins: (0) present; (1) absent.

30. Intraradial crossvein 4ir1: (0) present; (1) absent.

Note: This character was coded by Oswald as (0) for *Conchopterella* and *Micromus,* and as (1) for *Psectra*, although he states that it is polymorphic.

31. Posterodistal marginal silhouette: (0) convex; (1) concave.

Note: This character was coded by Oswald as (0) for *Conchopterella, Drepanepteryx, Hemerobius, Megalomina, Micromus and Sympherobius*, although he states that it is polymorphic.

32. Radiomedial crossvein 2r-m: (0) present; (1) absent.

Note: this character was coded by Oswald as (0) for *Notiobiella*, although he states that it is polymorphic.

33. Radiomedial crossvein 3r-m: (0) present; (1) absent.

Note: this character was coded by Oswald as (0) for *Micromus*, although he states that it is polymorphic.

34. Radiomedial crossvein 4r-m: (0) present; (1) absent.

Note: this character was coded by Oswald as (1) for *Notiobiella*, although he states that it is polymorphic. It was therefore here coded as polymorphic.

35. Intramedial crossvein 2im: (0) absent; (1) present

Note: this character was coded by Oswald as (0) for *Conchopterella, Megalomina* and *Micromus* although he states that it is polymorphic.

36. Intramedial crossvein 4im: (0) present; (1) absent.

37. Mediocubital crossvein 2m-cu: (0) present; (1) absent.

38. Mediocubital crossvein 3m-cu: (0) present; (1) absent.

Note: this character was coded by Oswald as (0) for *Hemerobius, Megalomus* and *Micromus*, although he states that it is polymorphic.

39. Mediocubital crossvein 4m-cu: (0) present; (1) absent.

Note: this character was coded by Oswald as (0) for *Micromus,* although he states that it is polymorphic.

40. Mediocubital flexion line: (0) present; (1) absent.

41. Intercubital crossvein 1cua-cup: (0) absent; (1) present.

Note: this character was coded by Oswald as (1) for *Neuronema*, although he states that it is polymorphic.

42. Depth of most proximal fork of forewing CuP: (0) fork absent or shallow, length of posterior CuP trace beyond fork > length of posterior CuP trace beyond fork; (1) deep, length of CuP stem before fork ≤ length of posterior CuP trace beyond fork.

Note: this character was coded by Oswald as (1) for *Conchopterella*, although he states that it is polymorphic.

43. Depth of most proximal fork of forewing A1: (0) absent or shallow, length of A1 stem before fork > length of posterior A1 trace beyond fork; (1) fork deep, length of A1 stem before fork ≤ length of posterior A1 trace beyond fork.

Note: this character was coded by Oswald as (1) for *Conchopterella* and as (0) for *Neuronema and Megalomus*, although he states that it is polymorphic.

44. 9^th^ tergite: (0) not completely divided sagittally (anterior and/or posterior margin may be sagittally emarginate); (1) completely divided sagittally.

45. Antecosta of 9^th^ tergite (lateral view): (0) not obliquely crossing tergite from anterior to posterior margin; (1) completely divided sagittally.

46. Cusp of posterolateral margins of male 9^th^ tergite: (0) absent; (1) present.

47. Posteroventral angle of 9^th^ tergite: (0) produced as a narrow membrane-margined lobe (Fig. 5A); (1) not prominently produced or, if somewhat produced, expanded as a broad membrane-margined lobe; (2) produced as an elongated subectoproctal lobe (usually) with a free distal process (Fig. 5B).

48. Horizontal costae of inner lateral surfaces of 9^th^ tergite: (0) absent; (1) present.

49. 9^th^ and 10^th^ tergites: (0) not fused (Fig. 5A); (1) fused (Fig. 5C).

50. 9^th^ sternite: (0) absent; (1) present, a free sclerite; (2) present, fused with 8^th^ sternite

51. Sagittal costa of dorsal surface of 9^th^ sternite: (0) absent; (1) present.

52. Apex of 9^th^ sternite (A): (0) not produced as a long, straight, attenuate tube; (1) produced as a long, straight, attenuate tube.

53. Apex of 9^th^ sternite (B): (0) not narrowly produced and abruptly upturned distally; (1) narrowly produced and abruptly upturned distally.

54. Digitiform posterior process of 9^th^ sternite: (0) absent; (1) present.

55. Posterior margin of 9^th^ sternite: (0) without a pair of acute cusps; (1) with a pair of acute cusps.

56. Posterolateral lobes of 9^th^ sternite: (0) absent; (1) present.

57. Paired elongate setae of 9^th^ sternite: (0) absent; (1) present.

58. Mesal surfaces of ectoprocts: (0) largely membranous (excluding narrowly protruding lobes); (1) largely sclerotized (narrowly protruding lobes absent).

59. Fusiform setae of ectoproct: (0) absent; (1) present.

60. Pecten of ectoproct: (0) absent; (1) present.

61. Articulated posteroventral lobe of ectoproct: (0) absent; (1) present.

62. Modified subapical seta of ectoproct: (0) absent; (1) present.

63. Sagittal emargination of posterior margin of extragonopons [not mediuncus]: (0) absent; (1) present.

64. Ventral margins of extrahemigonarcus: (0) not turned outward; (1) distinctly turned outward.

65. Horizontal costae of inner surfaces of intrahemigonarcus: (0) absent; (1) present.

66. Horizontal costae of outer surfaces of intrahemigonarcus: (0) absent; (1) present.

67. Lateral neogonarcal carinae of gonarcus: (0) absent; (1) present.

68. Gonofenestra: (0) absent; (1) present.

69. Gonofenestral plate: (0) absent (Fig. 5G); (1) present (Fig. 5H).

70. Secondary gonopons of gonarcus: (0) absent; (1) present.

71. Transverse sub basal costa of ventral surface of mediuncus: (0) absent; (1) present.

72. Apex of mediuncus: (0) mediuncus absent; (1) apex pointed or rounded (not emarginate); (2) emarginate; (3) terminating in a pair of long, slender, pliable processes.

73. Pseudomediuncus: (0) absent; (1) present (Fig. 5A).

74. Base of pseudomediuncus: (0) narrow (or pseudomediuncus absent); (1) laterally expanded

75. Pseudomediuncus divisions: (0) absent, pseudomediuncus absent or undivided; (1) present, divided intro two sclerites.

76. Gonosaccal membrane below mediuncus: (0) without a scabriculous region; (1) with a scabriculous region (Fig. 5D).

77. Phallolingua: (0) absent; (1) present.

78. Parabaculum (A): (0) absent; (1) present.

79. Parabaculum (B): (0) not deeply divided, or if deeply divided not as described for state 1 (Fig. 5E); (1) deeply (entirely or nearly entirely) divided, composed of a pair of adjacent, narrow sclerotized straps, which are enlarged posteriorly as small sclerotized surfaces surrounded by membrane, sagittal apophyseal lamella absent (Fig. 5F).

80. Sagittal division of parabaculum: (0) terminal cleft complete, parabaculum comprised of a pair of laterally adjacent sclerites; (1) terminal cleft incomplete (or parabaculum absent); (2) terminal cleft absent, parabaculum with a single undivided terminal lobe.

81. Dorsal subapical spinose processes of parabaculum: (0) absent; (1) present.

82. Ventrodistal region of parabaculum: (0) not expanded as a pair of rounded bulbous lobes (or parabaculum absent); (1) expanded as a pair of rounded bulbous lobes.

83. Bow in apophyseal shaft of parabaculum immediately anterior to terminal lobes: (0) absent; (1) present.

84. Penniform sclerites: (0) absent; (1) present.

85. Supra-penniform sclerite: (0) absent; (1) present.

86. Laterobacula: (0) absent; (1) present.

87. Gonosaccal accessory sclerite: (0) absent; (1) present.

88. Supragonopontal setal group: (0) absent; (1) present.

Note: this character was coded by Oswald as (1) for *Wesmaelius*, although he states that it is polymorphic.

89. 9^th^ gonocoxites: (0) present, articulating with or fused proximately to gonarcus; (1) absent.

90. 7^th^ sternite: (0) posterior margin sagittally emarginate; (1) not divided and posterior margin not emarginate; (2) sternite completely divided sagittally.

Note: this character was coded by Oswald as (0) for *Psectra*, although he states that it is polymorphic

91. Ventral margins of 9^th^ tergite: (0) without chalazate setae; (1) with chalazate setae

Note: this character was coded by Oswald as (0) for *Micromus*, although he states that it is polymorphic

92. Anteroventral lobes of 9^th^ tergite: (0) absent; (1) present.

93. 9^th^ gonocoxites: (0) membranously separated from ipsilateral 9^th^ tergite margins (Fig. 5I); (1) fused with ipsilateral 9^th^ tergite margins (Fig. 5J).

94. Styli of 9^th^ gonocoxites: (0) present (Fig. 5K); (1) absent (Fig.5I)

95. Sclerotized sulcus dividing 9^th^ gonocoxites: (0) absent; (1) present.

Note: this character was coded by Oswald as (0) for *Neuronema, Noius, Notiobiella* and *Psectra*, although he states that it is polymorphic.

96. Ventral lobes of 9^th^ gonocoxites: (0) absent; (1) present.

Note: this character was coded by Oswald as (0) for *Hemerobius*, *Megalomus* and *Micromus* although he states that it is polymorphic.

97. Gonapophyses posteriores: (0) present; (1) absent.

Note: this character was coded by Oswald as (0) for *Megalomus, Micromus, Notiobiella* and *Sympherobius*, although he states that it is polymorphic.

98. Insemination-fertilization canal: (0) slit-entry type; (1) pore-entry type.

99. Subgenitale: (0) present; (1) absent.

Note: this character was coded by Oswald as (0) for *Megalomus* and *Micromus,* although he states that it is polymorphic.

100. Sclerotization of female bursa: (0) bursa membranous, without sclerotized areas; (1) bursa broadly but weakly sclerotized dorsally; (2) bursa with lateral walls prominently sclerotized (3) bursa with a sclerotized dorsal arch transversely connecting lateral margins of 8^th^ sternite.

Note: this character was coded by Oswald as (0) for *Micromus, Neuronema* and *Notiobiella*, although he states that it is polymorphic.

101. Distal diverticulum of insemination-fertilization canal: (0) absent; (1) present.
